# Ascitic Senescent T Cells Are Linked to Chemoresistance in Patients With Advanced High-Grade Serous Ovarian Cancer

**DOI:** 10.3389/fonc.2022.864021

**Published:** 2022-07-07

**Authors:** Jie Zhang, Tianhui He, Zhongnan Yin, Chunliang Shang, Lixiang Xue, Hongyan Guo

**Affiliations:** ^1^Department of Obstetrics and Gynecology, Peking University Third Hospital, Beijing, China; ^2^Cancer Center, Peking University Third Hospital, Beijing, China; ^3^Center of Basic Medical Research, Institute of Medical Innovation and Research, Peking University Third Hospital, Beijing, China

**Keywords:** senescent T cells, HGSOC, ascites, chemosensitivity, biomarkers

## Abstract

Senescent T cells are reported to be increased in patients with cancer and are poor prognostic indicators. However, the distribution of senescent T cells and their correlation with clinical features in high-grade serous ovarian cancer (HGSOC) is unknown. We detected the percentage of senescent T cells in the peripheral blood and ascites of patients with advanced HGSOC (n = 86) at diagnosis by flow cytometry. Compared with healthy donors, patients with HGSOC exhibited an accumulation of CD28^−^CD57^+^ (Tsen) CD8^+^ T cells in the peripheral blood and ascites. The frequency of Tsen CD8^+^ T cells in the peripheral blood was positively correlated with age and pretreatment serum CA125 and increased in patients with large volume ascites, whereas the frequency of Tsen CD8^+^ T cells in ascites was elevated in patients with lymph node metastasis. Patients with Tsen-high in ascites (>19.92%), but not in the peripheral blood, were more likely to be resistant to chemotherapy and had shorter progression-free survival. Tsen CD8^+^ T cells exhibited common senescence features including increased SA-β-gal activity, declines in proliferation, loss of CD27 and gain of KLRG-1, and the production of cytokines. In ascites, the percentage of Tsen CD8^+^ T cells was positively correlated with levels of interleukin-10 and granzyme B. This study suggests the potential of ascitic Tsen CD8^+^ T cells at diagnosis as a prognostic biomarker in HGSOC.

## Introduction

Epithelial ovarian cancer is one of the most lethal gynecological diseases worldwide (1). High-grade serous ovarian cancer (HGSOC) is the most common and aggressive histologic ovarian cancer type (2). Most Patients with HGSOC are diagnosed at advanced stage (III/IV), accompanied by massive ascites and extensive abdominal metastasis (2). Tumor debulking surgery followed by platinum-based chemotherapy is the frontline treatment for patients with primary HGSOC. Nevertheless, most patients with HGSOC are at high risk of recurrence due to chemoresistance (3). Therefore, biomarkers are necessary to identify patients with poor prognosis and refine the treatment strategies for them.

Malignant ascites, an important feature of HGSOC, is known to facilitate metastasis and contribute to chemoresistance (4–6). This remarkable fluid is a common and easily available medical waste in patients with advanced HGSOC. In addition, the malignant ascites reflects both tumors and their microenvironment (4). This makes ascites an ideal material for lipid biopsy. High numbers of CD8^+^ effector memory T cells (5) and a low CD4/CD8 ratio (7) have been reported to be associated with longer progression-free survival (PFS). The high percentage of Tim-3^+^CD8^+^ T cells was associated with shorter overall survival (OS) (6). This indicates that patients’ prognosis is affected by the composition of immune cells in ascites.

Senescent T cells are commonly defined as CD28^−^CD57^+^ (8). They have unique phenotypes including the decreased level of costimulatory molecules CD27 and the increased level of CD45RA and KLRG-1 (8–10). They are defects in proliferation and activation but are capable to produce large amounts of cytokines (8). Several studies indicated that tumors evade immune surveillance by trigger the senescence of T cells (9–12). The pretreatment level of senescent T cells in the peripheral blood are reported to associate with outcomes in patients treated with chemotherapy (9). However, the results vary in different cancers. In gastric cancer (13), metastatic breast cancer (14), and acute myeloid leukemia (AML) (15), high levels of pretreatment senescent T cells in the peripheral blood correlated with short PFS and OS, whereas in non–small cell lung cancer (NSCLC), circulating senescent T cells were not associated with patients’ outcomes (16). The distribution of senescent T cells in the peripheral blood and ascites and the correlation between senescent CD8^+^ T cells and clinical features in HGSOC have yet to be determined.

In the present study, we investigated the presence of senescent CD8^+^ T cells in the peripheral blood and ascites from patients with newly diagnosed HGSOC and analyzed the relationship between senescent CD8^+^ T cells and clinical characters. We also explored phenotype and function of senescent CD8^+^ T cells. Our findings demonstrate that a high level of senescent CD8^+^ T cells associated with advanced disease and chemoresistance. This study suggests the potential of ascitic Tsen CD8^+^ T cells as prognostic biomarkers and therapeutic targets in HGSOC.

## Methods and Materials

### Study Design and Human Specimens

This study included patients with newly diagnosed high-grade serous ovarian, fallopian tube, or peritoneal cancer (admitted to the Peking University Third Hospital from May 2019 to December 2021). Patients with other types of benign or malignant ovarian diseases, general infection diseases, hematological diseases, renal diseases, liver diseases, and other tumors and those who received anti-tumor therapies, including surgery, chemotherapy, radiotherapy, and immunotherapy, during 5 years preceding enrollment, were excluded. Finally, 86 patients were eligible and included in our study. Fifty-three age- and sex-matched healthy donors (HDs) were chosen as control.

Basic characteristics of all HGSOC patients were collected, including age and serum cancer antigen 125 (CA125) levels at diagnosis. Staging was based on the International Federation of Gynecology and Obstetrics (FIGO) staging system (1). The outcome of the debulking surgery was defined as complete (no macroscopically visible residual tumor, R0), optimal (residual tumor foci ≤ 1 cm, R1), or suboptimal (residual tumor foci >1 cm, R2). R1 and R2 are collectively referred as NR0. The status of lymph node metastasis (LNM) was determined on the basis of postoperative pathological results. The best efficacy of the first-line treatment (complete remission, CR) was defined by normalized serum CA125 level, normal physical examination, and CT scan without evidence of recurrence (17). Patients who had not reach CR including partial remission, stable disease, and progressive disease were generally referred as not CR (NCR) (18). Patients with progression-free interval (PFI) < 6 months were termed as chemotherapy resistant, and those with PFI > 6 months were termed as chemotherapy sensitive. PFS was defined as period of time (months) from diagnosis to recurrence.

### Samples Collection, Processing, and Isolation of Lymphocytes

Pretreatment samples were collected before any anti-tumor therapy. Peripheral blood (8 ml) was collected from 53 HDs and 86 patients with HGSOC, stored in Ethylenediaminetetraacetic acid (EDTA) in anti-coagulant tubes. Twenty-five paired blood after neoadjuvant chemotherapy (NACT) and six paired blood after the whole chemotherapy cycles were also included. Seventy-three patients (84.88%) had ascites at diagnosis, and we obtained ascites fluid of 68 patients during surgery. All samples were treated within 1 h of collection.

Peripheral blood and ascites were centrifuged at 2,000 g for 10 min at 4°C. Mononuclear cells were obtained by density gradient centrifugation with Ficoll (1.077, GE, America) and phosphate buffer saline (PBS) at a ratio of 1:1.5. Samples were centrifuged at 400 g for 20 min without brake at 20°C. Cells were harvested and washed twice with PBS at 500 g for 5 min and counted manually.

### Flow Cytometry

For cell surface staining, cells (1 × 10^6^) from peripheral blood and ascites were stained with indicated monoclonal antibodies (mAbs) for 15 min in the dark at a room temperature. Subsequently, cells were fixed with 1% paraformaldehyde. Flow cytometric analysis was performed on CytoFLEX S (Beckman Coulter). Data were analyzed using Cytoexpert v. 2.3 software. All antibodies we used are listed in **Supplementary Table 1**.

### SA-β-Gal Staining

SA-β-gal activity in senescent T cells was detected using the Cellular Senescence Detection Kit (Dojindo Molecular Technologies, Gaithersburg, MD) according to the manufacturer’s instructions. Bafilomycin A1 was reconstituted in 30 μl of dimethyl sulfoxide, and SPiDER-βGal was reconstituted in 20 μl of dimethyl sulfoxide. Cells were then incubated with bafilomycin A-1 (1:500 dilution) for 1.5 h before the addition SPiDER-βGal (1:500 dilution) for another 30 min. Subsequently, cells were washed with PBS, stained with surface markers, and then measured by flow cytometry.

### Cell Proliferation Assay

Lymphocytes (1 × 10^7^ cells/ml) in ascites were incubated with 2 μM Carboxyfluorescein diacetate, succinimdyl ester (CFSE, BioLegend) at 37°C for 20 min. After washed with PBS, cells were cultured in RPMI 1640 medium (Hyclone) supplemented with 10% inactivated fetal calf serum (Gibco) and 1% penicillin/streptomycin (Hyclone) and subsequently stimulated with CD3 mAb (2 μg/ml; BioLegend) and CD28 mAb (1 μg/ml; BioLegend) for 72 h. Unstimulated PBMC were included as control. Cells were collected, and CFSE signal was measured by flow cytometry.

Alternatively, lymphocytes in ascites were directly stimulated with CD3 mAb (2 μg/ml; BioLegend) and CD28 mAb (1 μg/ml; BioLegend) for 72 h. Cells were collected, stained with Ki67, and measured by flow cytometry.

### Intracellular Cytokine Staining

For assessment of multiple cytokines production by CD8^+^ T cells, lymphocytes (3 × 10^5^ cells per well, 96-well plate) were cultured alone or with cell activation cocktail [phorbol-12-myristate-13-acetate (PMA) and ionomycin, BioLegend] and Brefeldin A (5 µg/ml, BioLegend) for 6 h. Cells were collected and stained with surface markers. After fixed and permeabilized using the Staining Buffer Kit (BioLegend), intracellular proteins were stained. Cells were washed and then acquired by flow cytometry.

### Cytokine Profiling by LEGENDplex™

Concentrations of 13 cytokines and cytotoxic molecules were analyzed in ascitic supernatant (n = 53) using the LEGENDplex™ Human CD8/NK Panel (13-plex, BioLegend). The assay was performed according to the manufacturer’s instructions. Flow cytometric analysis was performed on CytoFLEX S (Beckman Coulter). Data were analyzed using online software (BioLegend).

### Statistical Analysis

GraphPad Prism 9 and SPSS 23 were used for graphic representation and statistical analysis. All reported probability values were two-tailed, and a P value less than 0.05 was considered statistically significant. Statistical testing included t-test (data conformed to the normal distribution), Mann–Whitney U-test (data not conformed to the normal distribution), chi square (χ2) and Fisher’s exact tests, and Kaplan–Meier survival analysis with Gehan–Breslow–Wilcoxon test. Correlation between clinical characteristics or cytokines levels and frequency of Tsen CD8^+^ T cell was tested by linear regression (data conformed to the normal distribution) and Spearman’s rank coefficient (data not conformed to the normal distribution). Cutoff level (high vs. low) of Tsen was determined using the mean level of the cells. For multivariate analysis, the generalized linear model was used to analyze the independent influencing factors of Tsen. Multivariate Cox proportional hazards regression model was used to examine the independent risk factors for PFS.

### Study Approval

All sampling and experimental steps in this study were approved by the Ethics Committee of Peking University Third Hospital (License No. IRB00006761-M2019291).

## Results

### Demographics and Clinical Characteristics

Demographic and clinical characteristics of the included patients are summarized in **Table 1**. All patients with HGSOC were in stage III/IV. The median age of patients with HGSOC (n = 86) and HDs (n = 53) were both 56 years. Patients’ serum CA125 antigen level at diagnosis was 888.5 U/ml (21.09–19879.00 U/ml). Among 86 patients, 73 patients had ascites, including 23 patients with small volume ascites (<2,000 ml) and 50 patients with large volume ascites (>2,000 ml). Forty-six patients received primary debulking surgery (PDS), and 29 patients received ascites biopsy/biopsy surgery and two to four cycles of NACT, followed by interval debulking surgery (IDS). R0 was achieved in 40 of 75 patients. Eleven patients only received ascites biopsy/biopsy surgery due to poor performance and were excluded from the prognostic analysis. Three patients died after biopsy surgery, and two patients died after PDS; the remaining patients planned to receive four to eight cycles of platinum-based chemotherapy after debulking surgery. According to the postoperative pathological results, 36 patients had LNM and 27 patients had no LNM; the rest of the patients did not perform lymph node dissection. A good response to primary treatment (CR) was observed in 60 patients. Median PFS was 14.0 (4.0-36.0) months.

**Table 1:** Demographic characteristics and clinical parameters of study population. .

Characteristics	Peripheral Blood (n = 86)	Ascites(n = 68)
Age (median; interquartile range, years)	56 (32–85)	56 (32–85)
Serum CA125 level (median; interquartile range, U/ml)	888.50 (21.09–19879.00)	1149.00 (42.38–19879.00)
FIGO stage		
III	50 (58.14%)	39 (57.35%)
IV	36 (41.86%)	29 (42.65%)
Ascites		
Without	13 (15.12%)	–
<2,000 ml	23 (26.74%)	20 (29.41%)
>2,000 ml	50 (58.14%)	48 (70.59%)
Surgical procedure		
PDS	46 (53.49%)	30 (44.12%)
NACT+IDS	29 (33.72%)	28 (41.18%)
Ascites biopsy/biopsy surgery	11 (12.79%)	10 (14.71%)
Surgical satisfaction		
R0	40 (46.51%)	29 (42.65%)
NR0	35 (40.70%)	29 (42.65%)
No debulking	11 (12.79%)	10 (14.71%)
Lymph node metastasis		
Without	27 (31.40%)	19 (27.94%)
With	36 (41.86%)	27 (39.71%)
Unknown	23 (26.74%)	22 (32.35%)
Treatment efficacy		
CR	60 (69.77%)	46 (53.49%)
NCR	8 (9.30%)	7 (10.29%)
Under-treatment	4 (4.65%)	4 (5.88%)
Informal treatment	9 (10.47%)	7 (10.29%)
Dead before chemotherapy	5 (5.81%)	4 (5.88%)
Chemotherapy sensitivity		
Sensitive	43 (50%)	31 (45.59%)
Resistant	12 (13.95)	11 (16.18%)
Under-assessment	16 (18.60%)	14 (20.59%)
Informal treatment	9 (10.47%)	7 (10.29%)
Lost to follow-up	1 (1.16%)	1 (1.47%)
Dead before chemotherapy	5 (5.81%)	4 (5.88%)

CA125, cancer antigen 125; CR, complete response; FIGO stage, International Federation of Gynecology and Obstetrics stage; IDS, interval debulking surgery; NACT, neoadjuvant chemotherapy; NCR, not complete response; PDS, primary debulking surgery; R0, complete resection; NR0, incomplete resection.

### CD28^−^CD57^+^CD8^+^ T Cells Accumulated in the Peripheral Blood and Ascites From Patients With HGSOC

**Figure 1:**
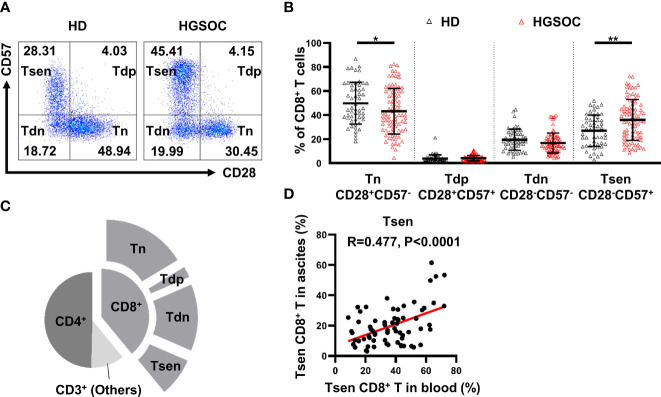
CD28^−^CD57^+^CD8^+^ T cells are present in the peripheral blood and ascites from untreated patients with HGSOC. **(A)** Representative flow cytometry plots are presented for CD28 and CD57 expression by CD8^+^ T cells in the peripheral blood of healthy donors (HDs, n = 53) and patients with high-grade serous ovarian cancer (HGSOC, n = 86). Values in plots represent percentages. **(B)** Proportion of CD8^+^ T-cell subsets: CD28^+^CD57^−^ (Tn), CD28^+^CD57^+^ (Tdp), CD28^−^CD57^−^ (Tdn), and CD28^−^CD57^+^ (Tsen). Groups were compared using t-test (Tsen) or Mann–Whitney U-test (Tn, Tdp, and Tdn). Bars show mean with SD. **(C)** Proportion of CD4^+^ and CD8^+^ subsets within CD3^+^ population in ascites (n = 68). **(D)** The correlation between the frequency of Tsen CD8^+^ T cells in the peripheral blood and ascites from the same patient with HGSOC (n = 68) was analyzed by Spearman’s rank coefficient. HD, healthy donors; HGSOC, high-grade serous ovarian cancer. *, P < 0.05; **, P < 0.01.

We first examined the presence of the main T-cell composition in the peripheral blood obtained from patients with HGSOC (n = 86) and age-matched HDs (n = 53). The proportion of total CD3^+^ T cells and CD8^+^ T cells were similar of HDs and HGSOC patients, and the frequency of CD4^+^ T cells was slightly elevated in patients with HGSOC (**Table 2**, **Supplementary Figure 1**). Loss of CD28 and gain of CD57 are prominent markers of senescent T cells (9, 10). Therefore, we used the markers CD28 and CD57 to identify four populations within CD8^+^ T cells: CD28^+^CD57^−^ (Tn), CD28^+^CD57^+^ (Tdp), CD28^−^CD57^−^ (Tdn), and CD28^−^CD57^+^ (Tsen). When compared with age-matched HDs, patients with HGSOC showed decreased percentage of Tn (43.16 ± 18.92% vs. 49.84 ± 17.34%, P = 0.039) and Tdn (16.74 ± 8.32 vs. 19.47 ± 8.82, P = 0.039) but increased percentage of Tsen (35.96 ± 17.01% vs. 26.95 ± 13.03%, P = 0.001) within CD8^+^ T cells in the peripheral blood (**Figures 1A, B**, **Table 2**). The percentage of Tdp was comparable between the two groups.

**Table 2:** Immunophenotyping of major T-cell subsets.

Markers (%)	Cell type	PB of HD (n = 53)	PB of HGSOC (n = 86)	Sig.	Ascites of HGSOC (n = 68)
CD3^+^ T	T	67.72 ± 8.78	68.81 ± 14.70	0.081^a^	72.66 ± 14.31
CD4^+^ T	Th	53.08 ± 13.95	57.68 ± 10.43	**0.042^b^**	49.99 ± 13.29
CD8^+^ T	Tc	35.54 ± 11.75	31.90 ± 9.71	0.151^a^	39.13 ± 12.25
CD8^+^CD28^+^CD57^−^ T	Tn CD8^+^ T	49.84 ± 17.34	43.16 ± 18.92	**0.039^b^**	41.69 ± 16.50
CD8^+^CD28^+^CD57^+^ T	Tdp CD8^+^ T	3.74 ± 3.12	4.14 ± 2.33	0.149^a^	5.49 ± 3.99
CD8^+^CD28^−^CD57^−^ T	Tdn CD8^+^ T	19.47 ± 8.82	16.74 ± 8.32	**0.039^a^**	32.90 ± 14.21
CD8^+^CD28^−^CD57^+^ T	Tsen CD8^+^ T	26.95 ± 13.03	35.96 ± 17.01	**0.001^b^**	19.92 ± 12.60

All data were shown as mean ± SD. ^a^ Data did not conform to normal distribution and were analyzed using the Mann–Whitney U-test. ^b^ Data conformed to normal distribution and were analyzed using t-test. P < 0.05 is considered significant. Statistically significant differences are highlighted in bold font.

Malignant ascites frequently develops in women with HGSOC (4, 19). The composition of T-cell subsets in ascites is associated with drug resistance and a poor prognosis (5, 6). In ascites from patients with HGSOC (n = 68), the proportion of CD4^+^ T (49.99 ± 13.29%) was greater than CD8^+^ T cells. Tn subsets (41.69 ± 16.50%) were predominant in CD8^+^ T cells, whereas 19.92 ± 12.60% of CD8^+^ T cells were Tsen (**Figure 1C**). Significantly positive correlation was found between the frequency of Tsen CD8^+^ T cells in the peripheral blood and ascites (**Figure 1D**).

Here, we reported that Tsen CD8^+^ T cells accumulated in the peripheral blood and ascites from patients with HGSOC at diagnosis.

### Tsen CD8^+^ T Cells Associated With Patients’ Clinical Characteristics

**Figure 2:**
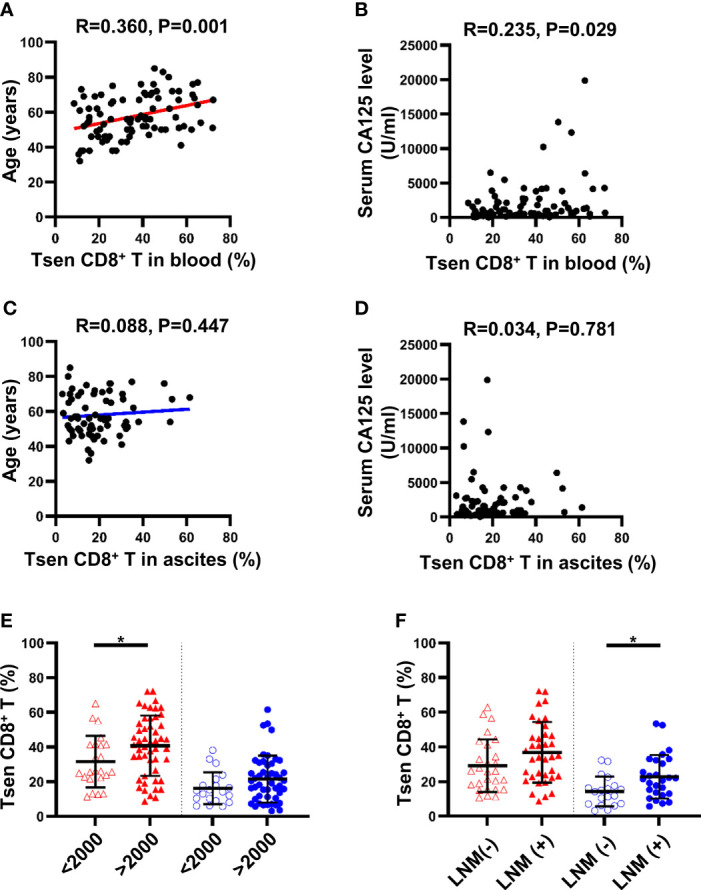
Clinical parameters and differences in proportion of Tsen CD8^+^ T cells in the peripheral blood and ascites of patients with HGSOC. The correlation between age and Tsen CD8^+^ T cells in **(A)** peripheral blood and **(C)** ascites was analyzed by linear regression test. Spearman’s rank coefficient was used to examine the correlation between serum CA125 level and Tsen CD8^+^ T cells in **(B)** peripheral blood and **(D)** ascites. Patients were grouped according to **(E)** ascites volume (peripheral blood: ascites volume <2,000 ml, n = 23, vs. ascites volume >2,000 ml, n = 50; ascites: ascites volume <2,000 ml, n = 20, vs. ascites volume >2,000 ml, n = 48) and **(F)** lymph node metastasis [peripheral blood: LNM (−), n = 27, vs. with LNM (+), n = 36; ascites: LNM (−), n = 19, vs. LNM (+), n = 27]. Groups were compared using t-test. Bars show mean with SD. *, P < 0.05.

We next investigated whether clinical characteristics of patients with HGSOC could be correlated to Tsen CD8^+^ T cells. In the peripheral blood, the percentage of Tsen CD8^+^ T cells was positively correlated with age (R = 0.36, P = 0.001) and pretreatment serum CA-125 levels (R = 0.235, P = 0.029) (**Figures 2A, B**). Patients with large volume ascites (>2,000 ml) had more frequency of Tsen CD8^+^ T cells in the peripheral blood compared with patients with low volume ascites (40.69 ± 17.42% vs. 31.49 ± 14.85%, P = 0.032; **Figure 2E**). The frequency of Tsen CD8^+^ T cells in the peripheral blood showed an increased tendency in patients with FIGO stage IV and LNM compared with those with FIGO stage III and without LNM (**Supplementary Figure 2**, **Figure 2F**).

Of the 86 peripheral blood, the mean percentage of Tsen in CD8^+^ T cells was 35.96%. Using the threshold of 35.96% to divide into Tsen-high and Tsen-low groups, 42 of 86 (48.84%) patients had high percentage of Tsen in the peripheral blood. In agreement with previous findings, pretreatment serum CA-125 levels, FIGO stage, and LNM were comparable between the two groups. However, patients in the Tsen-high group were older than patients in the Tsen-low group (**Table 3**). In addition, 73.81% (31/42) of patients with high Tsen CD8^+^ T cells had large volume ascites, whereas only 43.18% (19/44) of those in the Tsen-low group (P = 0.013, **Table 3**). Moreover, multivariate analysis revealed that age (P = 0.003), pretreatment serum CA-125 levels (P = 0.045), and ascites volume (P = 0.026) were independent risk factors of the frequency of Tsen in CD8^+^ T cells in the peripheral blood (**Table 4**).

**Table 3:** Clinical characteristics difference of patients with high/low levels of Tsen CD8^+^ T cells.

	Peripheral blood			Ascites		
	Tsen-low (<35.96%, n = 44)	Tsen-high (>35.96%, n = 42)	Sig.	Tsen-low (<19.92%, n = 39)	Tsen-high (>19.92%, n = 29)	Sig.
Age (median; interquartile range, years)	51 (32, 75)	63 (41, 85)	**<0.0001^a^**	56 (32, 85)	56 (41, 77)	0.27
Serum CA125 level (median; interquartile range, U/ml)	753.85 (21.09, 6503.00)	935.30 (51.15, 19879.00)	0.226^b^	1157.00 (46.21, 19879.00)	977.00 (274.00, 6402.00)	0.916
FIGO Stage			0.19			0.222
III	29	21		25	14	
IV	15	21		14	15	
ascites volume			**0.013**			0.066
without	10	3		–	–	
<2,000 ml	15	8		15	5	
>2,000 ml	19	31		24	24	
Lymph node metastasis			0.117			**0.033**
Without	20	7		25	4	
With	19	17		12	15	
Unknown^c^	5	18		12	10	

CA125, cancer antigen 125; FIGO stage, International Federation of Gynecology and Obstetrics stage. ^a^, Data conformed to normal distribution and were analyzed using t-test; ^b^, Data did not conform to normal distribution and were analyzed using the Mann–Whitney U-test; ^c^, This part of patients did not receive lymph node dissection and not include in the calculation. P < 0.05 is considered significant. Statistically significant differences are highlighted in bold font.

**Table 4:** Multivariate analyses for Tsen CD8^+^ T cells in the peripheral blood.

Variables	β (95%CI)	Sig.
Age	0.404 (0.139~0.668)	**0.003**
Serum CA125 level	0.001 (2.398E−5~0.002)	**0.045**
Ascites volume		**0.026**
without	0	
<2,000	4.850 −5.225~14.924)	0.345
>2,000	11.458 (2.287~20.630)	0.014

Multivariate analysis using generalized linear models. β, regression coefficients; CA125, cancer antigen 125; CI, confidence interval. P < 0.05 is considered significant. Statistically significant differences are highlighted in bold font.

In ascites, the percentage of Tsen CD8^+^ T cells showed no statistically correlation with age (R = 0.088, P = 0.477; **Figure 2C**) and pretreatment serum CA125 levels (R = 0.034, P = 0.781; **Figure 2D**). The frequency of ascitic Tsen CD8^+^ T cells showed an increased tendency in patients with FIGO stage IV and large volume ascites (>2,000 ml) compared with FIGO stage III and small volume ascites (**Supplementary Figure 2**, **Figure 2E**). Patients with LNM had more Tsen CD8^+^ T cells in ascites compared with patients without LNM (22.74 ± 12.58% vs. 14.25 ± 8.54%, P = 0.014; **Figure 2F**).

Of the 68 ascites, the mean percentage of Tsen in CD8^+^ T cells was 19.92%. Using the cutoff of 19.92% to divide patients into Tsen-high and Tsen-low groups, 29 of 68 (42.65%) patients had high percentage of Tsen in ascites. Most of clinical characteristics were similar in two groups (**Table 3**) except that LNM was significantly different between the two groups, with 12/39 (30.77%) in the low-Tsen group but 15/29 (51.72%) in the high-Tsen group (p = 0.033).

### Tsen Is a Negative Predictor of Chemotherapeutic Efficacy

Platinum-based chemotherapy is the frontline treatment for patients with primary HGSOC (1). To illustrate the influence of chemotherapy on Tsen CD8^+^ T cells, we compared the peripheral blood collected at diagnosis and that after NACT or the complete chemotherapy. The frequency of Tsen CD8^+^ T cells was not changed upon chemotherapy (**Supplementary Figure 3**). This indicated that circulating Tsen CD8^+^ T cells were not affected by chemotherapy.

**Figure 3:**
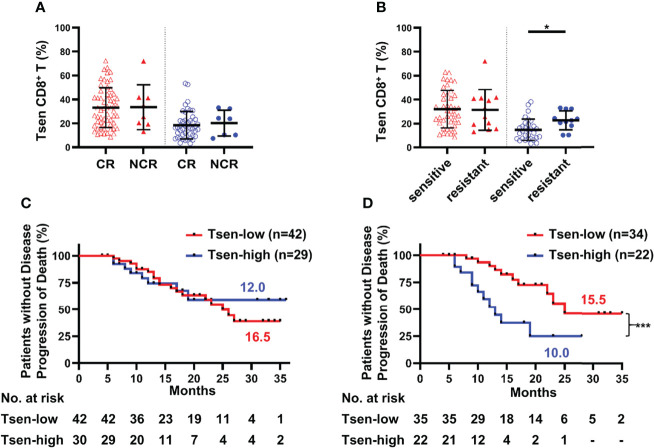
Tsen CD8^+^ T cells are a negative predictor of prognosis. Patients were grouped according to **(A)** CR/NCR (peripheral blood: CR, n = 60, vs. NCR, n = 8; ascites: CR, n = 46, vs. NCR, n = 7) and **(B)** chemotherapy sensitivity (peripheral blood: sensitive, n = 43, vs. resistant, n = 12; ascites: sensitive, n = 31, vs. resistant, n = 11). Groups were compared using t-test. Bars show mean with SD. The correlation of PFS and Tsen CD8^+^ T cells in **(C)** peripheral blood and **(D)** ascites at diagnosis were analyzed by Kaplan–Meier estimates (Gehan–Breslow–Wilcoxon test). Groups were made on the basis of mean values of the complete cohort. *, P < 0.05; ***, P < 0.001.

Complete remission after primary chemotherapy was widely accepted treatment efficacy evaluation index (17, 18). In our cohort, among 68 patients who received complete primary chemotherapy, 60 patients achieved CR (88.24%). No statistically difference was found between the percentage of Tsen in CR and NCR patient’ s blood or ascites (**Figure 3A**).

Chemosensitivity is an important predictor of survival in ovarian malignancies (20). Patients were divided into chemotherapy-sensitive and chemotherapy-resistant groups based on the length of PFI. Clinical characteristics were comparable between the two groups (**Supplementary Table 2**). In the peripheral blood, there was no difference in the frequency of Tsen CD8^+^ T cells in chemotherapy-resistant patients than those of chemotherapy-sensitive patients (31.96 ± 15.65% vs. 31.34 ± 16.97%; **Figure 3B**). However, significantly higher proportion of Tsen subset in CD8^+^ T cells was found in the ascites of chemotherapy-resistant patients than those of chemotherapy-sensitive patients (22.59 ± 7.93% vs. 14.68 ± 8.96%, P = 0.013; **Figure 3B**). In addition, the average PFI for the Tsen-high group was significantly shorter than the Tsen-low group (6.47 ± 5.89 months vs. 13.00 ± 8.00 months, P = 0.002).

Ultimately, we inquired whether Tsen CD8^+^ T cells in the peripheral blood and ascites could predict survival in patients with HGSOC. No correlation between PFS and the distribution of Tsen CD8^+^ T cells in the peripheral blood existed (**Figure 3C**). Of note, median PFS was significantly shorter for patients with the percentage of Tsen CD8^+^ T cells in ascites higher than 19.92% (10.0 months), compared with the Tsen-low group (15.5 months, P = 0.001; **Figure 3D**). Moreover, univariate analysis showed that patients with R0 (**Supplementary Figure 4D**) had better survival; however, surgical procedure (**Supplementary Figure 4A**), ascites volume (**Supplementary Figure 4B**), FIGO stage (**Supplementary Figure 4C**), and LNM (**Supplementary Figure 4E**) did not affect PFS in our cohort. Multivariate analysis revealed that the frequency of Tsen in CD8^+^ T cells in ascites was an independent risk factor of PFS (**Table 5**).

**Table 5:** Association of Tsen CD8^+^ T cells in ascites with progression-free survival in patients with advanced HGSOC.

Variables	No. of Patients	Hazard Ratio (95% CI)	Sig.
Surgical satisfaction			0.458
R0	33	1.00	
NR0	22	1.414 (0.566~3.534)	
Tsen CD8^+^ T cells			**0.016**
Low (<19.92%)	39	1.00	
High (>19.92%)	22	3.089 (1.239~7.703)	

Multivariate analysis using Cox proportional hazards regression. R0, complete resection; NR0, incomplete resection. P < 0.05 is considered significant. Statistically significant differences are highlighted in bold font.

### Tsen in HGSOC Ascites Exhibit Senescent Features

**Figure 4:**
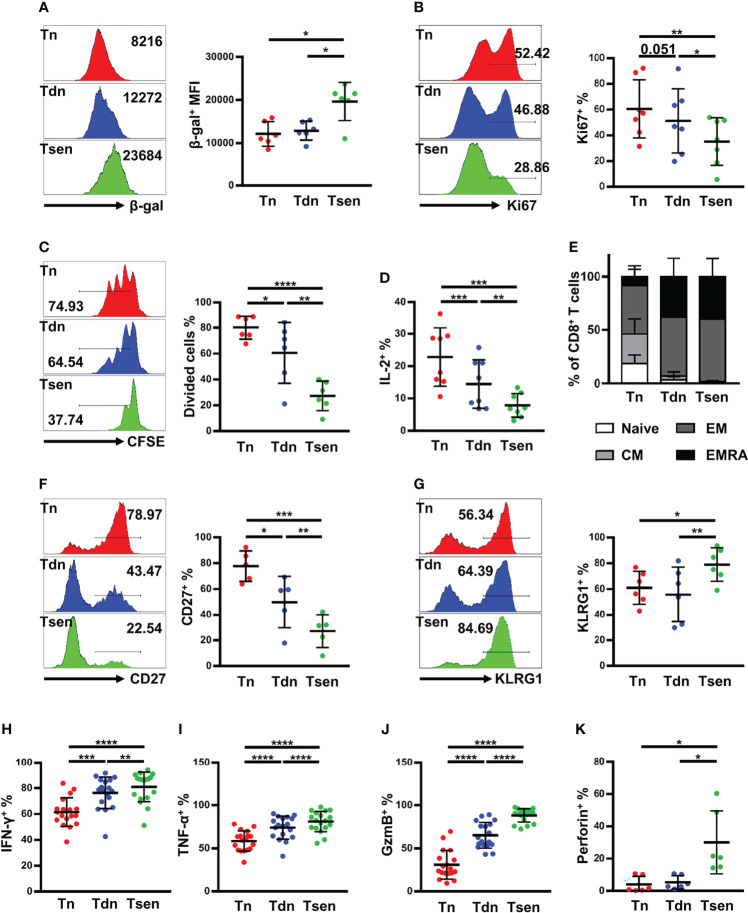
Ascitic Tsen CD8^+^ T cell in patients with HGSOC have a distinct senescent phenotype. The representative flow cytometry plots and the statistic diagram show **(A)** the mean fluorescence intensities (MFI) of SA-β-gal (n = 6); **(B)** the percentage of Ki-67^+^ (n = 7); **(C)** the level of dividing cells (n = 6); and **(D)** the percentage of IL-2^+^ (n = 8) in Tn, Tdn, and Tsen CD8^+^ T cells. **(E)** The statistic diagram shows the frequency of naive, CM, EM, and TEMRA (defined using the markers CCR7 and CD45RA) within Tn, Tdn, and Tsen CD8^+^ T cells (n = 22). The representative flow cytometry contour plots and the statistic diagram show the percentage of T-cell differentiation-associated markers **(F)** CD27^+^ (n = 5) and **(G)** KLRG1^+^ (n = 6) on Tn, Tdn, and Tsen CD8^+^ T cells. The representative flow cytometry plots and the statistic diagram show **(H)** IFN-γ (n = 18), **(I)** TNF-α (n = 18), **(J)** granzyme B (n = 18), and **(K)** perforin (n = 6) expression by Tn, Tdn and Tsen CD8^+^ T cells on stimulation with PMA/ionomycin for 6 h. Groups were compared using paired t-test **(A, C, D, F, G, I)** or Mann–Whitney U-test **(B, H, J, K)**. Bars show mean with SD.*, P < 0.05; **, P < 0.01; ***, P < 0.001; ****, P < 0.0001.

Next, we determined whether Tsen CD8^+^ T cells in ascites had other senescent features, such as high activity of SA-β-gal, cell cycle arrest, and other surface markers (9, 10). We found that Tsen CD8^+^ T cells in HGSOC ascites had the highest SA-β-gal activity compared with Tn and Tdn subsets (**Figure 4A**). Ki67 and CFSE staining were used to evaluate the proliferation capacity of CD8^+^ T cells in ascites from patients with HGSOC. After anti-CD3/CD28 stimulated for 3 days, Tsen CD8^+^ T cells showed lower frequency of Ki67^+^ cells and CFSE low cells (**Figures 4B, C**). Accordingly, the frequency of interleukin (IL-2) expression cells was significantly reduced in Tsen subsets compared with Tn and Tdn subsets (**Figure 4D**).

We also detect other surface makers on Tsen CD8^+^ T cells in ascites. Using the combination of CCR7 and CD45RA, CD8^+^ T cells can be defined into naïve [CCR7^+^CD45RA^+^, (N)], central memory [CCR7^+^CD45RA^−^, (CM)], effector memory [CCR7^−^CD45RA^−^, (EM)], and effector memory expressing CD45RA [CCR7^−^CD45RA^+^, (EMRA)]. We found that Tsen CD8^+^ T cells were predominantly EM or EMRA, whereas Tn CD8^+^ T cells were more naïve and CM cells (**Figure 4E**). Moreover, Tsen CD8^+^ T cells in ascites loss the expression of costimulatory molecules CD27 but gain the expression of KLRG-1 compared with Tn and Tdn subsets (**Figures 4F, G**).

Senescent T cells were reported to secrete lots of pro-inflammatory cytokines (9, 10). Lymphocytes in ascites were stimulated with PMA and ionomycin for 6 h, and intracellular staining for interferon-γ (IFN-γ) and tumor necrosis factor–α (TNF-α) was performed. The frequency of IFN-γ^+^ and TNF-α^+^ in Tsen CD8^+^ T cells was significantly higher than those in the paired Tn and Tdn subsets (**Figures 4H, I**). Granzyme B and perforin were also evaluated. We found that granzyme B and perforin were highly expressed in Tsen CD8^+^ T cells (**Figures 4J, K**).

### IL-10 and Granzyme B Correlated With the Accumulation of Tsen in Ascites

**Figure 5:**
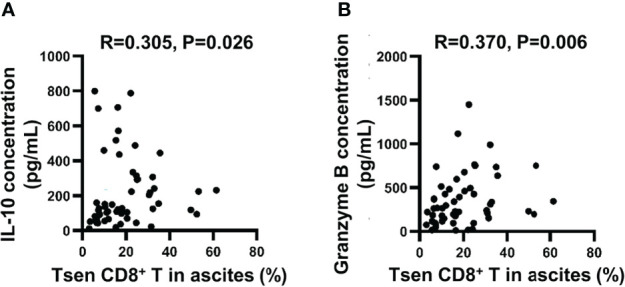
The correlation between ascitic cytokines and the percentage of Tsen CD8^+^ T cells. Correlation between **(A)** IL-10 or **(B)** granzyme B concentration and frequency of Tsen CD8^+^ T cells in ascites (n = 53) were tested using Spearman’s rank coefficient.

Finally, we analyzed the content of 13 soluble factors (IL-2, IL-4, IL-10, IL-6, IL-17A, TNF-α, sFas, sFasL, IFN-γ, granzyme A, granzyme B, perforin, and granulysin) in ascites with multiplex bead-based assay. All soluble factors with a median above the detection limit in ascitic supernatants are presented in **Table 6**. Consistent with a previous study (6), IL-6 was the most abundant factors in ascites. Among 13 soluble factors, increased IL-10 and granzyme B correlated with the accumulation of Tsen in ascites (**Figures 5A, B**).

**Table 6:** Ascites supernatants level of cytokines and their association with Tsen CD8^+^ T cells frequency in patients with HGSOC (n = 53).

Cytokines	Median (lower, upper)	Tsen CD8^+^ T cells	
		Correlation Coefficient	Sig.
IL-2	65.39 (1.94, 319.68)	0.233	0.093
IL-4	8.54 (0.27, 91.60)	0.206	0.139
IL-10	127.32 (11.60, 798.89)	0.305	**0.026**
IL-6	5003.02 (364.46, 1086575.76)	0.138	0.326
IL-17A	8.29 (2.22, 95.54)	0.130	0.353
TNF-α	8.07 (0.54, 118.56)	0.261	0.059
sFas	931.71 (181.13, 13687.67)	0.031	0.824
sFasL	12.52 (0.06-43.48)	-0.006	0.964
IFN-γ	29.19 (3.16, 409.76)	0.263	0.057
Granzyme A	80.40 (9.66, 381.27)	0.116	0.406
Granzyme B	265.75 (12.60, 1449.77)	0.370	**0.006**
Perforin	333.85 (71.12, 3480.11)	0.029	0.837
Granulysin	1571.48 (263.58, 10817.41)	0.132	0.346

IL, interleukin; IFN, interferon; TNF, tumor necrosis factor. P < 0.05 is considered significant. Statistically significant differences are highlighted in bold font.

## Discussion

T-cell senescence is a novel dysfunctional state in tumors (10). Senescent T cells elevate in patients with cancer and act as a potential biomarker to predict clinical outcomes (9). However, their role in HGSOC is unknown. We found that patients with untreated HGSOC exhibit an accumulation of Tsen CD8^+^ T cells in the peripheral blood and malignant ascites. The frequency of Tsen CD8^+^ T cells in the peripheral blood was positively correlated with age and pretreatment serum CA125 and increased in patients with large volume ascites. The frequency of Tsen CD8^+^ T cells in ascites was elevated in patients with LNM. Furthermore, patients with Tsen-high ascites (>19.92%) were more likely to resistant to chemotherapy and had shorter PFS. Our results point to the pretreatment frequency of ascitic Tsen CD8^+^ T cells as a biomarker of worse clinical outcomes.

Senescent T cells increased in the peripheral blood of patients with solid tumor (13, 21–24) and hematologic malignancies (25, 26). In line with previous studies, patients with HGSOC showed elevated frequency of Tsen CD8^+^ T cells in the peripheral blood compared with age-matched HDs. In patients with breast cancer, the frequency of Tsen CD4^+^ T cells was also significantly increased (27). However, in patients with HGSOC, the frequency of Tsen CD4^+^ T cells in the peripheral blood was comparable to HDs (data not shown). Therefore, in this study, we focused on the correlation of Tsen CD8^+^ T cells with clinical characteristics and outcomes of patients with HGSOC.

Integrating the above findings with the clinical data, we demonstrated that high abundance of Tsen CD8^+^ T cells in the peripheral blood was associated with increasing age, pretreatment serum CA125, and ascitic volume, whereas in ascites, the accumulation of Tsen CD8^+^ T cells was related to LNM. In line with previous study, Tsen frequency elevated in late stage in NSCLC (28). These findings suggested that Tsen CD8^+^ T cells might be an indicator of advanced disease. On one hand, because senescent T cells lose their capacity for antitumor immunity, patients with higher Tsen CD8^+^ T cells may experience more aggressive tumors. This idea might be supported by that old age (29) and HMCV infection (30), which exacerbate the senescence of T cells, may promote ovarian cancer progression. With a defect in T-cell receptor (TCR) signaling (31, 32) and TCR diversity (33), senescent T cells might be difficult to proliferate and activate upon tumor antigens stimulation (9). Interestingly, senescent T cells can function like natural killer (NK) cells to kill tumor cells independent of TCR by secreting granzyme B and perforin (32). It seems like that the expression of NK cell-like functions in CD8^+^ T cells could be an adaptation that would maintain antitumor effect to some extent. Senescent T cells are also able to produce inflammatory cytokines (IFN-γ and TNF-α) to modulate the tumor microenvironment. Consistent with the previous findings (34), the frequency of IFN-γ^+^ and TNF-α^+^ in Tsen CD8^+^ T cells was significantly higher than those in the paired Tn and Tdn subsets. Increased levels of IFN-γ and TNF-α would promote resistance to anti-tumor treatments by inducing tumor cell stemness (35) and the expression of immune suppressive factors (36). Therefore, the cytotoxicity of senescent CD8^+^ T cells should be further determined.

On the other hand, aggressive tumor may trigger the senescence of T cells. In the tumor microenvironment, the mechanisms and signaling pathways responsible for the induction of T-cell senescence remained unclear. Tumor cells would initiate DNA damage in effector T cells resulting in T-cell senescence and function changes (12, 37). IL-10, which was positively correlated with the level of Tsen CD8^+^ T cells in ascites (**Figure 5A**), was reported to induce senescence of hepatic stellate cells (38). In addition, glucose supplement (37) and inhibition of lipid droplet accumulation (12) may also suppress the senescence of T cells to restore antitumor effect. Less Tsen CD8^+^ T cells in patients with HGSOC was associated with better survival; hence, therapeutic approaches to prevent T-cell senescence are warranted. Potentially, targeting IL-10 and metabolic regulation in the tumor microenvironment may be of help.

The percentage of Tsen CD8^+^ T cells in ascites positively correlated with that in the peripheral blood. However, their relevance to clinical characteristics were quite different. In the peripheral blood, age, pretreatment serum CA125 levels, and ascitic volume were independent factors of Tsen CD8^+^ T cells, whereas in ascites, LNM was related to Tsen CD8^+^ T cells. This is probably because the malignant ascites may better reflect both tumors and their microenvironment than the peripheral blood did. In the peripheral blood, the percentage of Tsen CD8^+^ T cells was also affected by age and chronic infections (8). Ascites is known to facilitate metastasis and contribute to chemoresistance (4). The loss of immune surveillance of ascitic Tsen CD8^+^ T cells may further promote tumor metastasis. It is worth noting that the function of Tsen CD8^+^ T cells in the peripheral blood and tumors might be diverse. In patients with NSCLC, CD57^+^ T cells at the tumor site were much more defected in the cytokine production and proliferation compared with that in the peripheral blood (21, 39). However, in our study, we did not detect Tsen in the tumor tissue. An important question is whether blood and ascitic biomarker reflects what is going on in the tumor microenvironment. The peripheral blood, ascites, and tumor tissues collected from same patients are needed to evaluate this question.

Most patients with HGSOC will relapse or develop metastases despite a high initial response rate to surgery and chemotherapy (3). Therefore, biomarkers to predict chemotherapy efficacy remains a challenge. Several studies demonstrated that high levels of pretreatment senescent T cells in the peripheral blood correlated with short PFS and OS in gastric cancer (13), metastatic breast cancer (14), AML (15), and NSCLC (28). In contrast, our cohort and another NSCLC cohort (16) reported that the level of pretreatment Tsen in the peripheral blood was irrelevant to patients’ chemosensitivity or PFS. This may indicate the high heterogeneity of Tsen CD8^+^ T cells in various cancers. Meanwhile, we found that high pretreatment Tsen CD8^+^ T cells (>19.92%) in ascites was able to predict chemoresistance and shorter PFS in patients with advanced HGSOC. Potentially, preventing or rejuvenating senescence in CD8^+^ T cells may improve the efficacy of chemotherapy if Tsen CD8^+^ T cells are high in patients with stage III/IV HGSOC. It is worth noting that chemotherapy was reported to induce T-cell senescence (40–42). However, we found no impact of chemotherapy on the percentage of circulating Tsen CD8^+^ T cells (**Supplementary Figure 3**). In addition, we did not include relapsed patients’ ascites and blood to exclude another driving factor in the ascites of chemotherapy-resistant patients.

Immune checkpoint blockade is novel for ovarian cancer treatment; however, the response rate is limited (43). The antitumor activity of pembrolizumab was modest in patients with recurrent or newly diagnosed advanced ovarian cancer even if the expression of programmed cell death-Ligand 1 (PD-L1) was high (44–48), suggesting that other tumor immune-evasive mechanisms remain in HGSOC. In this study, we found accumulated Tsen CD8^+^ T cells correlated with advanced disease, sensitivity, and survival to chemotherapy. Interestingly, the invigoration of exhausted CD8^+^ T cells by programmed cell death protein1 (PD-1)/PD-L1 inhibition depends on CD28 signaling (49, 50). *In vitro* experiment showed that blocking PD-1 had no effect on the proliferation of functionality of CD28^−^CD8^+^ T cells and CD57^+^CD8^+^ T cells (21, 51). In advanced NSCLC and melanoma, patients with a high level of circulating Tsen CD8^+^ T cells exhibited poor response rate and short PFS (16, 52). Indeed, it may be reasonable to consider the senescence of CD8^+^ T cells as a novel immunological mechanism associated with immunotherapy resistance in HGSOC. Preventing or rejuvenating of Tsen CD8^+^ T cells may complement other therapeutic options in advanced HGSOC.

Our data should be interpreted considering some limitations. First, the sample size was limited, and our clinical studies were conducted in a single institution. To draw definitive conclusions, a multi-institutional investigation is warranted. Second, because of the lack of ascites and blood samples from relapsed patients, it is hard to exclude the role of factors other than Tsen CD8^+^ T cells in chemotherapy resistant. Third, the biological mechanisms of resistance to chemotherapy involving Tsen CD8^+^ T cells need to be clarified.

In summary, a high level of Tsen CD8^+^ T cells in blood and ascites showed positive correlation with advanced disease. Ascitic Tsen CD8^+^ T cells exhibited senescent profile and were relevant to chemoresistance and short PFS in patients with advanced HGSOC. Our results highlight the potential of Tsen CD8^+^ T cells as prognostic biomarkers and therapeutic targets in HGSOC.

## Data Availability Statement

The original contributions presented in the study are included in the article/**Supplementary Material**. Further inquiries can be directed to the corresponding authors.

## Ethics Statement

The studies involving human participants were approved by the Ethics Committee of Peking University Third Hospital (License No. IRB00006761-M2019291). The patients/participants provided their written informed consent to participate in this study.

## Author Contributions

LX and HG contributed to the concept development and study design. JZ, TH, and ZY performed the laboratory studies. TH and CS collected the clinical data. JZ and TH contributed to data analysis and figure preparation and drafted the manuscript. All authors read and approved the final manuscript.

## Funding

This work was supported by Beijing-Tianjin-Hebei Basic Research Cooperation Project (J200015), Ningxia Hui Autonomous Region’s key research and development plan (2019BFG2002-02), Youth program of Beijing Municipal Natural Science Foundation (7204328), and Key Clinical Projects of Peking University Third Hospital (BYSYZD2019034).

## Conflict of Interest

The authors declare that the research was conducted in the absence of any commercial or financial relationships that could be construed as a potential conflict of interest.

## Publisher’s Note

All claims expressed in this article are solely those of the authors and do not necessarily represent those of their affiliated organizations, or those of the publisher, the editors and the reviewers. Any product that may be evaluated in this article, or claim that may be made by its manufacturer, is not guaranteed or endorsed by the publisher.

## Abbreviations

AML, acute myeloid leukemia; CA125, cancer antigen 125; CFSE, Carboxyfluorescein diacetate, succinimdyl ester; CR, complete remission; EDTA, Ethylenediaminetetraacetic acid; FIGO, International Federation of Gynecology and Obstetrics; HDs, healthy donors; HGSOC, high-grade serous ovarian cancer; IDS, interval debulking surgery; IFN, interferon; IL, interleukin; LNM, lymph node metastasis; mAbs, monoclonal antibodies; NACT, neoadjuvant chemotherapy; NCR, not complete remission; NK, natural killer; NSCLC, non–small cell lung cancer; OS, overall survival; PBS, phosphate buffer saline; PD-1, programmed cell death protein 1; PD-L1, programmed cell death-Ligand 1; PDS, primary debulking surgery; PFI, progression-free interval; PFS, progression-free survival; PMA, phorbol-12-myristate-13-acetate; TCR, T-cell receptor; TNF, tumor necrosis factor; Tregs, regulatory T cells.
