# Supplementary material for: Ascitic Senescent T Cells Are Linked to Chemoresistance in Patients With Advanced High-Grade Serous Ovarian Cancer
Source: Front Oncol. 2022 Jul 7;12:864021. doi: 10.3389/fonc.2022.864021 (PMC9301961; doi:10.3389/fonc.2022.864021)
Supplement: Supplementary file 1 [file DataSheet_1.docx]

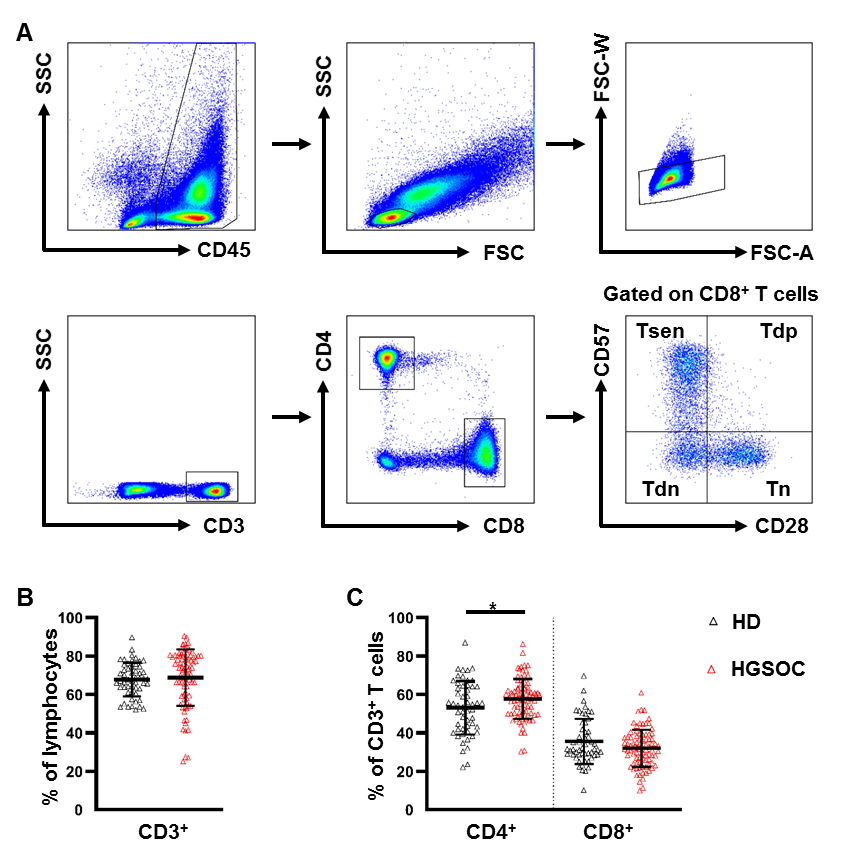


Supplementary Figure 1. Characterization of T cell composition in peripheral blood of healthy donors (HD, n = 53) and HGSOC patients (n = 86). (A) Representative plots from all presented populations are shown. Proportion of (B) CD3^+^ T cells (gated from lymphocytes), (C) CD4^+^ and CD8^+^ T cells (gated from CD3^+^ T cells). *, P < 0.05.


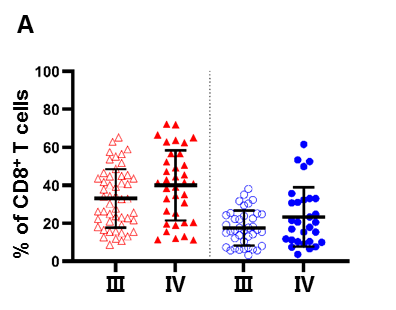


Supplementary Figure 2. The correlation between FIGO stage and the frequency of Tsen CD8^+^ T cells in peripheral blood (n = 86) and ascites (n = 68) from HGSOC patients. Patients were grouped according to FIGO stage (peripheral blood: Ⅲ, n=50 vs Ⅳ, n=36; ascites: Ⅲ, n=39 vs Ⅳ, n=29) and compared using t test. Bars show mean with SD.


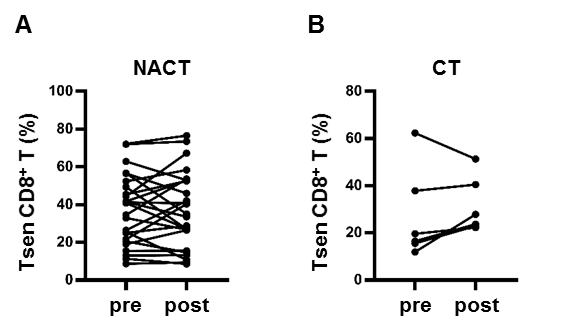


Supplementary Figure 3. Proportions of Tsen CD8^+^ T cells in peripheral blood of HGSOC patients at (A) pre-treatment and post-NACT (n = 25), and (B) pre-treatment and post-frontline treatment (n = 6).


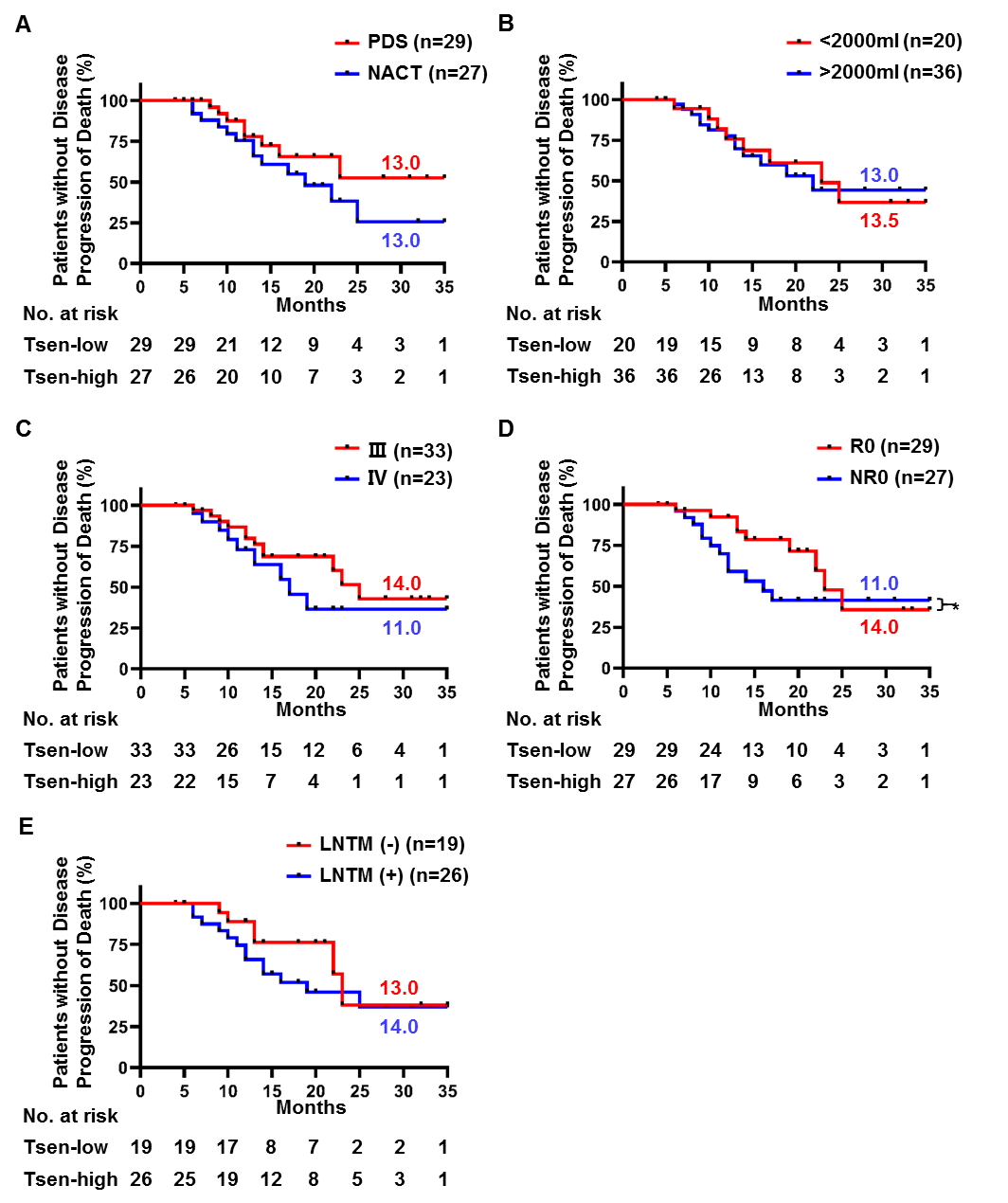


Supplementary Figure 4. Univariate analyses for survival of HGSOC patients. The correlation of PFS and (A) surgical procedure, (B) ascites volume, (C) FIGO stage, (D) surgical satisfaction, (E) lymph node metastasis in HGSOC patients with ascites specimens were analysed by Kaplan–Meier estimates (Gehan-Breslow-Wilcoxon test). Groups were made based on mean values of the complete cohort. *, P < 0.05.

Supplementary Table 1 Reagent used in this research

| Reagent | Com | Catalog | Clone |
| --- | --- | --- | --- |
| FITC anti-human CD3 | Biolegend | 300306 | HIT3a |
| APC-Cy7 anti-human CD3 | Biolegend | 300426 | UCHT1 |
| APC-Cy7 anti-human CD4 | Biolegend | 300426 | A161A1 |
| PE anti-human CD4 | Biolegend | 300508 | RPA-T4 |
| PerCP-cy5.5 anti-human CD8 | Biolegend | 344710 | SK1 |
| PE-Cy7 anti-human CD28 | Biolegend | 302926 | CD28.2 |
| BV605 anti-human CD28 | Biolegend | 302968 | CD28.2 |
| Brilliant Violet 605™ anti-human CD45 | Biolegend | 304041 | HI30 |
| FITC anti-human CD57 | Biolegend | 359604 | HNK-1 |
| Pacific Blue anti-human CD57 | Biolegend | 359607 | HNK-1 |
| PE anti-human PD-1 | Biolegend | 329906 | EH12.2H7 |
| PE-CF594 anti-human CCR7 | Biolegend | 353236 | G043H7 |
| BV510 anti-human CD45RA | Biolegend | 304141 | HI100 |
| BV421 anti-human Tim-3 | Biolegend | 345007 | F38-2E2 |
| BV650 anti-human Lag-3 | Biolegend | 369315 | 11C3C65 |
| PE-CF594 anti-human KLRG1 | Biolegend | 367709 | SA231A2 |
| APC anti-human CD27 | Biolegend | 302809 | O323 |
| APC anti-human Ki67 | Biolegend | 350514 | Ki-67 |
| FITC anti-human IFN-γ | Biolegend | 502506 | 4S.B3 |
| PE-CF594 anti-human TNF-α | Biolegend | 502946 | MAb11 |
| APC anti-human Granzyme B | Biolegend | 372204 | QA16A02 |
| PE anti-human Perforin | Biolegend | 308105 | dG9 |
| BV605 anti-human IL-2 | Biolegend | 500331 | MQ1-17H12 |
| BV650 anti-human CD107a | Biolegend | 328637 | H4A3 |
| Ultra-LEAF™ Purified anti-human CD3 | Biolegend | 300331 | HIT3a |
| Ultra-LEAF™ Purified anti-human CD28 | Biolegend | 302934 | CD28.2 |
| Cell Activation Cocktail (without Brefeldin A) | Biolegend | 423302 | - |
| Brefeldin A Solution (1,000X) | Biolegend | 420601 | - |
| CFSE Cell Division Tracker Kit | Biolegend | 423801 | - |
| LEGENDplex™ Human CD8/NK Panel (13-plex) | Biolegend | 740267 | - |
| Fixation Buffer | Biolegend | 420801 | - |
| Intracellular Staining Perm Wash Buffer (10X) | Biolegend | 421002 | - |
| True Nuclear^TM^ 4X Fix Concentrate | Biolegend | 73158 | - |
| True Nuclear^TM^ Fix Diluent | Biolegend | 73160 | - |
| True Nuclear^TM^ 10X Perm | Biolegend | 73162 |  |
| Cellular Senescence Detection Kit | Dojindo | SG03 | - |

Supplementary Table 2 Univariate analyses for chemosensitivity of HGSOC patients with ascites (n = 42)

|  | sensitive (n = 31) | resistant (n = 11) | Sig. |
| --- | --- | --- | --- |
| Age (median; interquartile range, years) | 53.19 ± 10.68 | 57.09 ± 6.83 | 0.177^a^ |
| Serum CA125 level (median; interquartile range, U/mL) | 2368.25 ± 4096.39 | 1747.88 ± 1780.49 | 0.955^b^ |
| Surgical procedure |  | | 0.159 |
| PDS | 18 | 3 |  |
| NACT+IDS | 13 | 8 |  |
| FIGO Stage |  | | 0.281 |
| III | 21 | 5 |  |
| IV | 10 | 6 |  |
| Ascites volume |  | | 1.000 |
| < 2000 ml | 11 | 4 |  |
| > 2000 ml | 20 | 7 |  |
| Surgical satisfaction |  | | 0.159 |
| R0 | 18 | 3 |  |
| NR0 | 13 | 8 |  |
| Lymph node metastasis |  | | 0.711 |
| Without | 12 | 3 |  |
| With | 16 | 6 |  |
| Unknown^c^ | 3 | 2 |  |

Univariate analysis using Chi-square test. FIGO stage, International Federation of Gynecology and Obstetrics stage; IDS, interval debulking surgery; NACT, neoadjuvant chemotherapy; PDS, primary debulking surgery; R0, complete resection; NR0, incomplete resection. ^a^, The data did not conform to normal distribution and were analyzed using Man Whitney U test; ^b^, the data conformed to normal distribution and were analyzed using t test; ^c^, this part of patients did not receive lymph node dissection and not include in the calculation. P < 0.05 is considered significant.
